# Twist angle-dependent conductivities across MoS_2_/graphene heterojunctions

**DOI:** 10.1038/s41467-018-06555-w

**Published:** 2018-10-04

**Authors:** Mengzhou Liao, Ze-Wen Wu, Luojun Du, Tingting Zhang, Zheng Wei, Jianqi Zhu, Hua Yu, Jian Tang, Lin Gu, Yanxia Xing, Rong Yang, Dongxia Shi, Yugui Yao, Guangyu Zhang

**Affiliations:** 1 CAS Key Laboratory of Nanoscale Physics and Devices, Institute of Physics, Chinese Academy of Sciences, Beijing, 100190 China; 2 School of Physical Sciences, University of Chinese Academy of Sciences, Beijing, 100190 China; 3 Beijing Key Laboratory of Nanophotonics and Ultrafine Optoelectronic Systems, School of Physics, Beijing Institute of Technology, Beijing, 100081 China; 4 Beijing Key Laboratory for Nanomaterials and Nanodevices, Beijing, 100190 China; 5 Collaborative Innovation Center of Quantum Matter, Beijing, 100190 China

## Abstract

Van der Waals heterostructures stacked from different two-dimensional materials offer a unique platform for addressing many fundamental physics and construction of advanced devices. Twist angle between the two individual layers plays a crucial role in tuning the heterostructure properties. Here we report the experimental investigation of the twist angle-dependent conductivities in MoS_2_/graphene van der Waals heterojunctions. We found that the vertical conductivity of the heterojunction can be tuned by ∼5 times under different twist configurations, and the highest/lowest conductivity occurs at a twist angle of 0°/30°. Density functional theory simulations suggest that this conductivity change originates from the transmission coefficient difference in the heterojunctions with different twist angles. Our work provides a guidance in using the MoS_2_/graphene heterojunction for electronics, especially on reducing the contact resistance in MoS_2_ devices as well as other TMDCs devices contacted by graphene.

## Abstract (web summary)

Twisting vertically stacked individual layers of two-dimensional materials can trigger exciting fundamental physics and advanced electronic device applications. Here, the authors report five times enhancement in vertical heterojunction conductivity on rotating MoS_2_ over graphene.

## Introduction

Two-dimensional (2D) materials can be assembled into the so-called Van der Waals (vdW) heterostructures, offering an exotic platform for exploring many fundamental physics and important device applications^1,2^. One of the crucial structural parameters in a vdW heterostructure is its twist angle, a new degree of freedom for modulating its properties. Indeed, many fascinating twist angle-dependent properties have been investigated including the strong twist angle-dependent resistivity of Gr/graphite contact^3,4^, the quantum transport in Graphene/BN superlattice^5–7^, resonant tunneling in Gr/BN/Gr^8^, interlayer excitons in MoSe_2_/WSe_2_^9^, and band evolution in MoS_2_/graphite^10^, just to mention a few. Among many investigations, unraveling the vertical conductivity of a vdW heterojunction is of fundamental importance; however, only limited knowledge has been established so far.

Here, we report the experimental investigation of vertical conductivities of MoS_2_/graphene (MoS_2_/Gr) heterojunctions under various lattice twist configurations. We found that vertical conductivities of these heterojunctions are strongly twist angle-dependent. When varying the twist angle from 0° to 30°, conductivities monotonically decrease and the modulation depth (maximum/minimum) is ∼5. Considering that MoS_2_/Gr heterojunction has great potential in various devices applications^11,12^ and even batteries^13^, graphene can also form excellent contact to MoS_2_^14–17^; our results provide a guidance in using the MoS_2_/Gr heterojunction for electronics.

## Results

### Growth and characterization of MoS_2_/Gr heterojunction

**Fig. 1:**
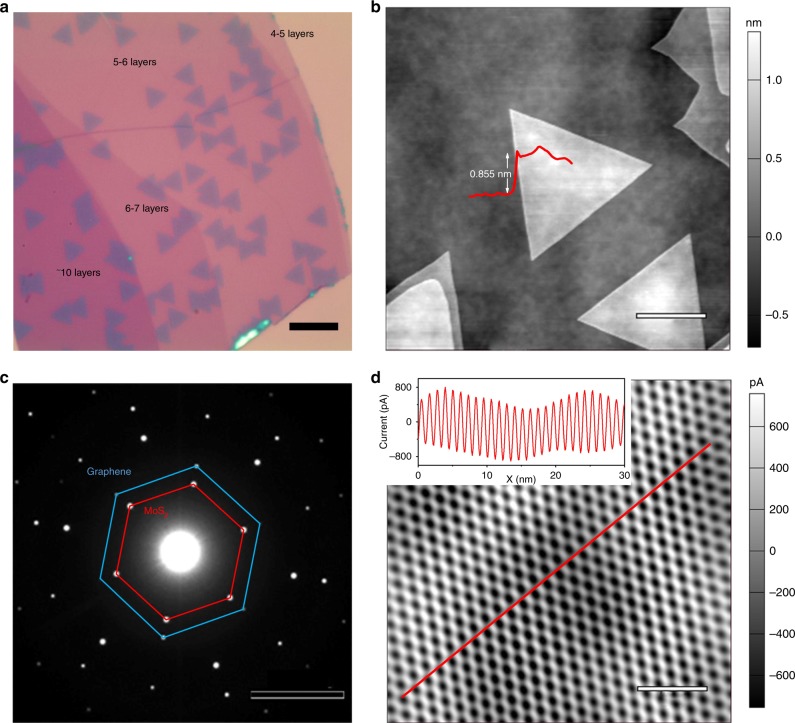
Structures of MoS_2_/Gr heterojunctions. **a** Optical image of as-grown MoS_2_ triangle domains on graphene, scale bar, 10 μm. **b** AFM image, scale bar, 2 μm. **c** SAED pattern of a typical MoS_2_/Gr heterojunction, scale bar, 5 nm^−1^. **d** C-AFM imaging of the moiré superlattice of MoS_2_/Gr after Fast Fourier Transform filter, scale bar, 5 nm. The period of superlattice is ∼1.18 nm as shown in the inset

In this study, the MoS_2_/Gr heterojunctions were fabricated by an epitaxial growth technique^18,19^ (also see Methods for more details). Figure 1a shows an optical image of as-grown MoS_2_ triangle domains on graphene. These domains have obviously preferred orientations and similar sizes, suggesting the epitaxy nature of MoS_2_ domains. Figure 1b shows the atomic force microscope (AFM) image of an area zoomed in Fig. 1a. It can be clearly seen that the surfaces of both MoS_2_ and graphene substrate are clean and free of contaminations. The height of as-grown MoS_2_ triangle domains is ∼0.855 nm, corresponding a monolayer thickness^20,21^. Raman and photoluminescence (PL) spectra also indicate that the monolayer MoS_2_ domains are of high qualities (Supplementary Figure 1). Selected area electron diffraction (SAED) was also used to characterize the lattice alignment of our MoS_2_/Gr samples. As illustrated in a typical pattern (Fig. 1c), the hexagonal diffraction spots of both MoS_2_ and graphene have the same orientations, suggesting an either 0° or 60° twist angle, which are geometrically equivalent between the as-grown MoS_2_ and graphene. To further demonstrate the high quality and the clean surface of these MoS_2_ domains, we further performed conductive AFM (C-AFM) imaging (Fig. 1d and Supplementary Figure 2). We can clearly see that the moiré superlattice with a period of ∼1.18 nm arises from the lattice mismatch between MoS_2_ and graphene (∼30%)^22^. Note that in Fig. 1c, the sample is very thin, leading to quite a large center transmission spot, and diffraction spots from the moiré superlattice are not visible in the SEAD pattern, since they are within the center transmission spot.

### Twist-angle-dependent conductivities of MoS_2_/Gr heterojunction

**Fig. 2:**
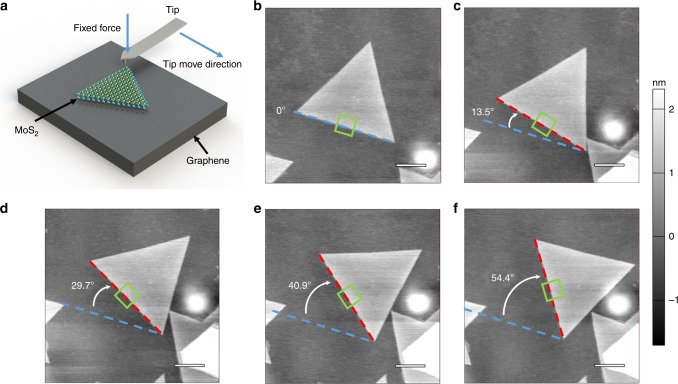
Rotation of as-grown MoS_2_ domains on graphene substrates. **a** Schematic of AFM-tip manipulation setup. **b**–**f** AFM images of a typical MoS_2_ domain rotated on graphene to achieve a series of twist angles, scale bar, 1 μm. Blue dash lines indicate the original direction of the MoS_2_ domain, while white arrows indicate the rotation directions. Green rectangles represent the scan area during C-AFM measurements

To achieve different twist angles between the MoS_2_ domains and the graphene substrates underneath, we rotated these as-grown MoS_2_ domains via AFM-tip manipulation technique. This manipulation process is like that reported in our previous work^18,23^. As illustrated in Fig. 2a, during the rotation process, we firstly engaged the AFM tip to the graphene surface with a load of tens of nN and then pushed the MoS_2_ triangle from its corner to actuate its rotation. By controlling the tip moving direction and length, we thus can rotate the MoS_2_ domains on graphene with any deterministic twist angles, as illustrated in Fig. 2b–f. The heights of this MoS_2_ domain under different twist angles are likely to be different if considering the interlayer coupling; however, due to the probing limit (no more than 60 pm) of our AFM, these height variations were not observed in our experiments.

**Fig. 3:**
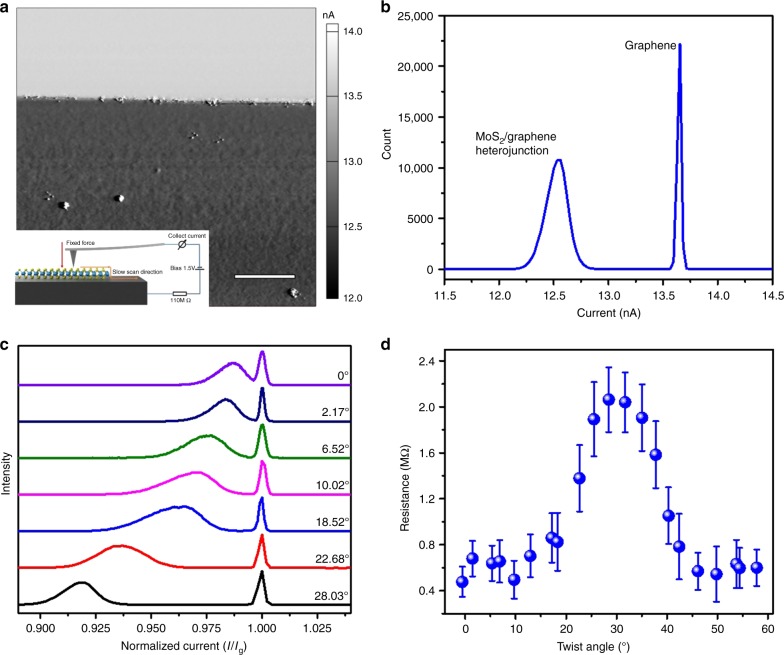
Twist-angle-dependent conductivities of MoS_2_/Gr heterojunctions. **a** A typical current mapping of a MoS_2_/Gr heterojunction with a twist angle of ∼28.03°, scale bar, 100 nm. Bright and dark areas in this mapping reflect the tip-to-sample current through the bare graphene and heterojunction, respectively. Inset is the experimental setup for current mapping. **b** Statistical chart derived from **a**. **c** Normalized current distributions of seven heterojunctions with different twist angles. **d** Statistic resistances of MoS_2_/Gr heterojunctions with different twist angles. Error bar is derived from the full width at half maximum of the MoS_2_ peaks, etch statistics contains at least 20,000 points

Since MoS_2_/Gr heterostructures with deterministic twist angles are available, we thus measured their vertical conductivities to investigate the twist-angle dependence. In our experiments, the conductivity was directly measured under the C-AFM mode. During C-AFM scanning, a metal-coated AFM tip was placed in direct contact with the sample surface under a controlled load of ∼23 nN (cantilevers were calibrated by Sader’s method)^24^. The bias applied to the measurement circuit was fixed to be 1.5 V, and a 110-MΩ resistor was connected into the circuit to prevent current overload to our C-AFM holder, which has a measuring range of ±20 nA and a noise level of 1.5 pA, as illustrated in the inset of Fig. 3a. Note that, during scanning, we adjusted the fast scan direction of the AFM tip in parallel to one edge of the MoS_2_ triangle to reduce the feedback noise.

Figure 3a shows a typical C-AFM current mapping of a MoS_2_/Gr heterojunction with a twist angle of 28.03°. The mapping can be clearly divided into two different regions: the bright region from the bare graphene surface with higher conductivities and the dark region from the MoS_2_/Gr heterojunction with lower conductivities. To quantitatively extract the conductivity meanwhile eliminating the metal at the two different regions, we thus inverted this current mapping into a statistical current distribution chart as illustrated in Fig. 3b. The *x*-axis of the chart is current and the *y*-axis is the counts, which is from an area of 500 nm × 500 nm, corresponding to 256 × 256 points. From the statistics, we can clearly see two Gaussian peaks whose centers reflect the statistic currents *I*_g_ and *I*_j_ flowing through the graphene and the junction, respectively. Because the resistance of the graphene is much smaller than the connected 110-MΩ resistor, *R*_g_ = *V/I*_g_ just reflects the system resistance and barely shifts. In order to facilitate comparison, we used *Q* *=* *I*_j_/*I*_g_ to normalize the current distribution statistics.

Figure 3c shows the normalized current distribution statistics for a series of MoS_2_/Gr heterojunctions with different twist angles *θ*. The intensity comes from the scale of heterojunction and graphene area we chose to make statistics. It can be clearly seen that the two peaks are moving apart to each other when the MoS_2_/Gr heterojunction is twisted from 0° to 30°. As illustrated in Fig. 3d, we also calculated the resistances of the MoS_2_/Gr heterojunctions with different twist angles, which shows an obvious 60° period. (Details of resistance calculation, please see Supplementary Note 5) The resistance at *θ* *=* 30° is about 5 times larger than that at *θ* *=* 60° (0°). Error bar in Fig. 3d is derived from the full width at half maximum of the MoS_2_ peaks, which can characterize the dispersion of the heterojunction resistance. From the results, we can see that the dispersion of the heterojunction resistance has no clear correlation with twist angles. In addition to the measurements of these MoS_2_/Gr heterojunctions fabricated by AFM-tip-facilitated rotation, we also measured a polycrystalline MoS_2_/Gr heterojunction (Supplementary Figure 5), in which different twist angles naturally exist in an individual sample. Current mapping also shows bright and dark areas clearly separated by the 30° grain boundaries, consistent with the above observations. Note that we reproduced the above experiments many times on different samples. In each time we at least captured five current maps for an individual twist angle, and the overall results show good consistency.

### Theorical simulations

As observed above, vertical conductivities across the MoS_2_/Gr heterojunctions are highly twist-angle dependent. Since the tip is in direct contact with the sample surface during C-AFM scanning and tip-to-MoS_2_ interface is not likely to have any lattice orientation dependent effects, this interface-modulated conductivities could be related to the stacking configurations between graphene and MoS_2_. The tunneling current can be described as^25–27^:

1 $$I\left( V \right) \propto {\int} {{d}E \cdot DoS_{\mathrm{g}}} \left( E \right) \cdot DoS_{\mathrm{t}}\left( {E - eV} \right) \\ \cdot \left[ \, {f\left( {E - eV} \right) - f\left( E \right)} \right] \cdot T\left( E \right)$$

where $DoS_{\mathrm{g}}\left( E \right)$ and $DoS_{\mathrm{t}}\left( {E - eV} \right)$ are the available tunneling density of states in graphene and the metal tip. *f*(*E*) is the Fermi–Dirac distribution. *T*(*E*) is the transmission coefficient. It can be seen that the tunneling current is dominated by the transmission coefficient under a certain tip-to-sample bias.

**Fig. 4:**
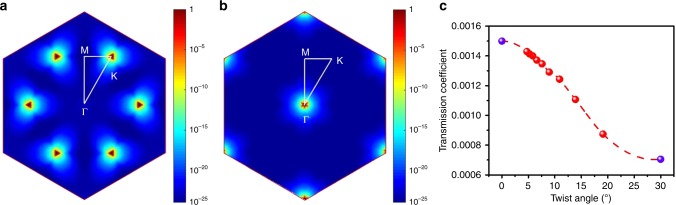
Theorical simulations. **a**, **b** Simulated K-space transmission hot mappings for the 0°- and 30°-twisted MoS_2_/Gr heterojunctions. **c** Transmission coefficient as a function of twist angles. Red points are calculated by WKB method and blue points are calculated by DFT

To unravel the variation of transmission coefficient in the MoS_2_/Gr heterojunctions, we thus performed density functional theory (DFT) simulations (please see Methods and Supplementary Note 7). For simplicity, we just calculated the transmission coefficients for *θ* *=* 0° and *θ* *=* 30° configurations and got *T*_0_ = 0.0015 and *T*_30_ = 0.000705, respectively. Two numbers are in the ratio 2.21:1, suggesting the tunneling current of the 0°-twisted heterojunction will be 2.21 times larger than that of the 30°-twisted heterojunction under a same bias. This result is in fair agreement with our observations, although our experimental results indicate that the ratio should ∼5. This experiment theory inconsistency might come from choosing structural parameters used for simulations, considering that little variation of structural parameters could lead to significant change on the simulated results. In our model, the periodic symmetry has been broken along the transport direction, but preserved on the flat straighten to the transport direction. As a result, a 2D Brillouin zone exists. Figure 4a and b show the simulated hot mappings of transmission coefficients in K-space for the 0°- and 30°-twisted heterojunctions, respectively, providing a hint on which momentum area dominates the transport mostly. It can be clearly seen that K points dominate transport mostly for the 0°-twisted heterojunction; in sharp contrast, Γ points contributes to transport mostly for the 30°-twisted heterojunction. In order to give a clearer relationship between the twist angle and the transmission coefficient, we also applied Wentzel–Kramers–Brillouin (WKB) method to generate transmission data under different twist angles as shown in Fig. 4c. We can clearly see that the transmission coefficient decreases monotonically as twist angles varying from 0° toward 30°, consistent with our experiment results. (Please see Methods for more details of WKB calculations.)

Considering the evolution of MoS_2_/Gr heterojunctions with twist angles from 0° to 30°, we can simply treat them by rotating the Brillouin zone of graphene. At *θ* *=* 0°, K points for graphene and MoS_2_ are aligned. During rotating, graphene’s K points will move away from MoS_2_’s K points; at *θ* *=* 30°, graphene’s K points are at MoS_2_’s Γ points due to the band folding. From K point to Γ point, there is a k-path along which MoS_2_’s bandgap changes from minimum to maximum. As a result, the transmission coefficient of this system changes from the largest to the smallest, which is consistent with the above experimental observations shown in Fig. 3d.

## Discussion

We investigated MoS_2_/Gr heterojunctions with different twist angles in terms of their vertical conductivities. We found that the vertical conductivities of these heterojunctions can be modulated up to ∼5 times by twist angles with the highest/lowest conductivity occurs at a twist angle of 0°/30°. DFT simulations suggest that this twist-angle-dependent conductivities originates from the transmission coefficient difference in the MoS_2_/Gr heterojunctions. Our work provides a guidance in using the MoS_2_/Gr heterojunction for electronics, especially concern on reducing the contact resistance in MoS_2_ devices as well as other TMDCs devices contacted by graphene.

## Methods

### Sample preparations

The MoS_2_ growth was performed in a three-temperature-zone chemical vapor deposition (CVD) chamber. S (Alfa Aesar, 99.9%, 4 g) and MoO_3_ (Alfa Aesar, 99.999%, 50 mg) sources were loaded in two separate inner tubes and placed at zone-I and zone-II, respectively. Substrates were load in zone-III. During the growth, Ar/O_2_ (gas flow rate: 75/3 sccm) are used as carrying gases and temperatures for the S-source, MoO_3_-source, and substrates are 115 °C, 530 °C, and 930 °C, respectively. Thin graphene flakes were mechanically exfoliated from both HOPG and Graphenium graphite (Manchester Nanomaterials) and placed on SiO_2_ substrates.

### Sample characterizations

PL and Raman characterizations were performed in a Horiba Jobin Yvon LabRAM HR-Evolution Raman system using a 532-nm laser excitation wavelength. SAED was performed in a TEM (Philips CM200) operating at 200 kV.

### AFM and C-AFM scanning

AFM imaging was performed by Asylum Research Cypher S with AC160TS tip. In C-AFM mode, we used ASYELEC-01 tip with Ti/Ir coating and the holder 901.730. The bias was applied from sample to tip with a current limit of ±20 nA and noise of 1.5 pA.

### DFT calculations

We used DFT within the Keldysh non-equilibrium Green’s function (NEGF) formalism^28^. Succinctly, NEGF-DFT calculates density matrix by NEGF as ${\rho \sim {\int } {\mathrm d}EG^{ < }}$, and transmission coefficient as $T = T_{r}\left[ {G^{r}\Gamma _LG^{a}\Gamma _R} \right]$. While, $G^{r,a, < }$ are the retarded, advanced, and lesser Green’s functions, respectively, and *Γ*_*L*,*R*_ are the self-energy of the left (L) and right (R) leads. Furthermore, the conductance $G = T \cdot e^2{\mathrm{/}}h$, where *e* is the electron charge and *h* is the Planck constant. In our model. We choose multilayer graphene for both left lead and right lead, considering the lattice match problem. The structure has mirror symmetry centered on the plane of molybdenum atoms. The structure parameters have been chosen as: superlattice constant: 1.2816 nm; thickness of MoS_2_: 0.3227 nm (0°) and 0.3174 nm (30°); interlayer spacing of MoS_2_/Gr heterojunction: 0.3110 nm (0°) and 0.3131 nm (30°); interlayer spacing of graphene: 0.34 nm, all same with ref. ^29^.

### WKB calculations

In WKB calculations, MoS_2_ was treated as a limited barrier; thus, the transmission coefficient has a simple relation with the twist angle, described as $T = ae^{ - b\sqrt {E_{\mathrm{g}}} }$. Where, *a*, *b* are pending constants, *T* is transmission, and *E*_g_ is the bandgap. Since we already have two transmissions hot maps for two twist angles, i.e., 0° and 30°, which have been used to determine *a* and *b* in the equation. In order to obtain the relation between the tunneling gap and the twist angle, band-folding technique has been used.

## Electronic supplementary material

Supplementary Information

## Data Availability

The authors declare that the data supporting the findings of this study are available within the paper and its supplementary information files.
