# Supplementary material for: Twist angle-dependent conductivities across MoS2/graphene heterojunctions
Source: Nat Commun. 2018 Oct 4;9:4068. doi: 10.1038/s41467-018-06555-w (PMC6172227; doi:10.1038/s41467-018-06555-w)
Supplement: Supplementary file 1 — Supplementary Information [file 41467_2018_6555_MOESM1_ESM.pdf]

Supplementary information of
Twist angle dependent conductivities across MoS_2/graphene heterojunctions

Mengzhou Liao et al.

Supplementary information of Twist angle dependent conductivities across MoS_2/graphene heterojunctions

Mengzhou Liao[1,2†], Zewen Wu[3†], Luojun Du[1,2], Tingting Zhang[1,2,3], Zheng Wei[1,2], Jianqi Zhu[1,2], Hua Yu[1,2], Jian Tang[1,2], Lin Gu[1,2], Yanxia Xing[3], Rong Yang[1,2,4], Dongxia Shi[1,2,4], Yugui Yao[3*] and Guangyu Zhang[1,2,4,5*]

[1] *CAS Key Laboratory of Nanoscale Physics and Devices, Institute of Physics, Chinese Academy of Sciences, Beijing 100190, China*
[2] *School of Physical Sciences, University of Chinese Academy of Sciences, Beijing 100190, China*
[3] *Beijing Key Laboratory of Nanophotonics and Ultrafine Optoelectronic Systems, School of Physics, Beijing Institute of Technology, Beijing 100081, China*
[4] *Beijing Key Laboratory for Nanomaterials and Nanodevices, Beijing 100190, China*
[5] *Collaborative Innovation Center of Quantum Matter, Beijing 100190, China*

[†] Authors contributed equally to this work.

* Corresponding authors. E-mail: gyzhang@aphy.iphy.ac.cn; ygyao@bit.edu.cn

Supplementary Notes

Supplementary Note 1: Raman and photoluminescence spectra of MoS_2/Gr heterostructure

From the Raman spectra in Supplementary Figure 1a, we can see the peak of E_{2g} is ~ 385cm^{-1} and the peak of A_{1g} is ~ 407cm^{-1}. The distance between the two peaks is 22 cm^{-1}, indicating monolayer MoS_2. In PL spectra, we cannot see any feature arising from the emission of defect-trapped excitons, shows the heterojunction is of good quality and with low defect densities.

Supplementary Note 2: moiré superlattice of MoS_2/Gr heterojunction

During the AFM scanning, we could directly see moiré superlattice of pristine MoS_2/Gr heterojunctions. The period of the superlattice is ~1.18 nm, agree with pervious works. The emergence of moiré superlattice indicates both good quality of our sample and the high resolution of our AFM system.

Supplementary Note 3: AFM-tip facilitated manipulation process

We use a special model in our AFM (Cypher S by Asylum Research) called Lithography mode, which we can program tip route under a specific pressure and speed. We capture pictures both before and after manipulation process, by comparing these two images, we then could determinate the twist angle of the heterostructure.

Supplementary Note 4: I/V curves of MoS_2/Gr heterojunction and graphene

We have measured the I/V curves at the heterojunction or graphene areas. The local bias is the actual bias between tip and sample. Fig. S3a is the I/V curve of 0° twist angle heterojunction and Supplementary Figure 3b is the I/V curve of graphene. It is clear the I/V curve of graphene is linear and the I/V curve of heterojunction has a platform near zero bias.

Here we use tunneling model to estimate the barrier height of the junction. Similar with pervious works we direct treat the MoS_2 layer as a simple finite barrier[1,2]. For simplification we just consider the tunneling near Fermi level and assume the available tunneling density of states in graphene and the metal tip is constant and Fermi-Dirac distribution will not change under small bias. Thus the tunneling current near the Fermi level is dominated by the transmission coefficient $I_{E_F}(V_b) \propto T_{E_F}(V_b)$. We use same formula of $T_{E_F}(V_b)$ as ref. 26 in the main text to fit the small bias region of Supplementary Figure 3a, results are shown in Supplementary Figure 3c and d. These fittings work very well at the low bias case according this tunneling model. The resulted barrier heights at negative/positive bias regions are ~0.428/0.205eV. For higher bias, the fittings are bad, means the barrier cannot be treated as a simple finite barrier as well as our assumptions are no longer valid under high bias condition.

Supplementary Note 5: Calculations on the resistance

In the experiment the bias applied on the circuit was fixed to 1.5V, if the current flowed through the heterojunction area is I_j, then the total resistance of the circuit is $R_t = V/I_j$. if the resistance of the measurement system (which includes the series 110-MΩ resistor and internal resistance of AFM circuit) is R_s, then the resistance of heterojunction could be calculated by $R_j = R_t - R_s$. Because the resistance of the graphene is much smaller than the connected 110MΩ resistor, $R_g = V/I_g$ just reflect the system resistance and barely shift. So $R_s = R_g$ and the resistance of heterojunction $R_j = V/I_j - V/I_g$.

Note that, during the C-AFM measurements, the bias voltages between tip and graphene is proportional to the resistance of the heterojunction due to the series 110-MΩ resistor. It means for higher heterojunction resistance the sample-to-tip bias is lager and sample-to-tip bias are different under different twist angles. Actually, the bias of ~30° twist angle is ~5 times larger than 0° twist angle. According to ref.26, the tunneling current is exponential relationship to the bias. Thus, the variation of resistance is underestimated, the resistance difference between 0° and 30° twist angles shall be even larger if bias keeps same.

In Supplementary Figure 4a, we show the raw current maps of Fig. 3d with different twist angles. In the current maps we can clear see the heterojunction region becomes darker when twist angle closer to 30° while the graphite region is barely changed at any twist angles. Supplementary Figure 4b we show data in Fig.3c before normalization. Supplementary Figure 4c shows the Gauss fitting of the heterojunction and graphite peaks, it shows the current is Gaussian distribution. To demonstrate the repeatability of our consequence, in Supplementary Figure 4d we give another run on a different sample which the heterojunction has been twisted more than one period. In Supplementary Figure 4e, we gave three consecutive captured current maps of 10.32°, which shows the variation is low.

Supplementary Note 6: AFM image of polycrystalline MoS_2 on graphene

The current mapping of a polycrystalline MoS2/Gr heterojunction naturally with 0° and 30° twist-angles components in Supplementary Figure 5c shows bright and dark areas clearly separated by the 30° grain boundaries. The resistance of 30° area is larger than the 0° area, consistent with our previous observations.

The growth process of polycrystalline MoS_2 on graphene is almost the same with that for single crystalline samples but in a 2-inch growth system and on Kish graphene substrate at 800 centigrade. Compared with natural graphene we use, Kish graphene has much more stages and prefer to grow polycrystalline MoS_2. Polycrystalline MoS_2

can also be seem on our natural graphene, but relatively rare.

Supplementary Note 7: Details on the DFT calculations.

We used Density Functional Theory (DFT) within the Keldysh non-equilibrium Green's function (NEGF) formalism to study this Gr/MoS_2 heterostructure. Supplementary Figure 6a-c shows the Gr/MoS_2/Gr atomic structure in our analysis. For predigestion, we choose multilayer graphene for both left lead and right lead, considering the lattice match problem. The structure has mirror symmetry centered on the plane of molybdenum atoms. All the structure parameters have been chosen as the same with ref.[3]. We simulated two structures with different twist angles between graphene and MoS_2, as shown in Supplementary Figure 6b and c. All calculations have been done with Nanodcal package based on NEGF-DFT. Besides, norm-conserving pseudopotential is used to describe core electrons, as well as double-ζ polarization (DZP) linear combination of atomic orbital (LCAO) is used to describe valence electrons. The exchange correlation is treated by local density approximation (LDA).

As shown in Supplementary Figure 6d-e, the band structures of the graphene lead in two atomic structures: for 0° twist angle, there is only state around K point at fermi level; and for 30° twist angle, there is only state around Γ point at fermi level due to the band-folding. Moreover, it is the state around Fermi level contributes to transport. Obviously, for 0° twist angle structure, it will tunnel through MoS_2 at K point where the smallest band gap. As a consequence, the transmission coefficient is the biggest. But for 30° atomic structure, incoming state in graphene is at Γ point, which has a bigger band gap. As a consequence, the transmission coefficient is smaller.

Supplementary Figures

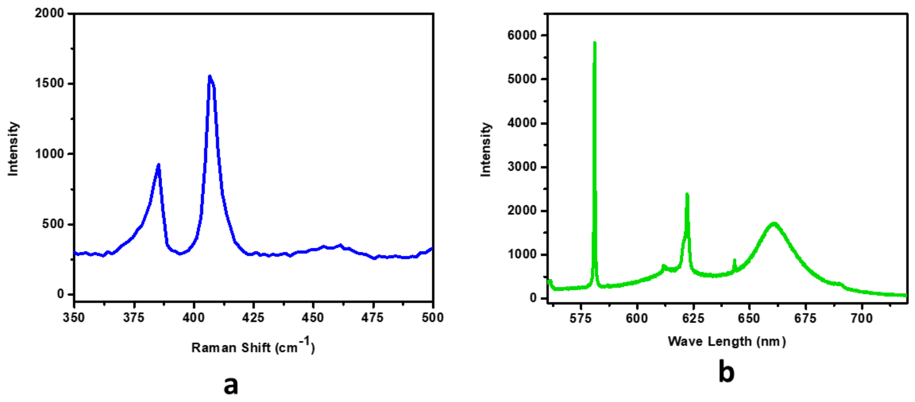

Supplementary Figure 1 | **(a)** Raman spectra and **(b)** PL spectra of as-grown MoS_2 on graphene.

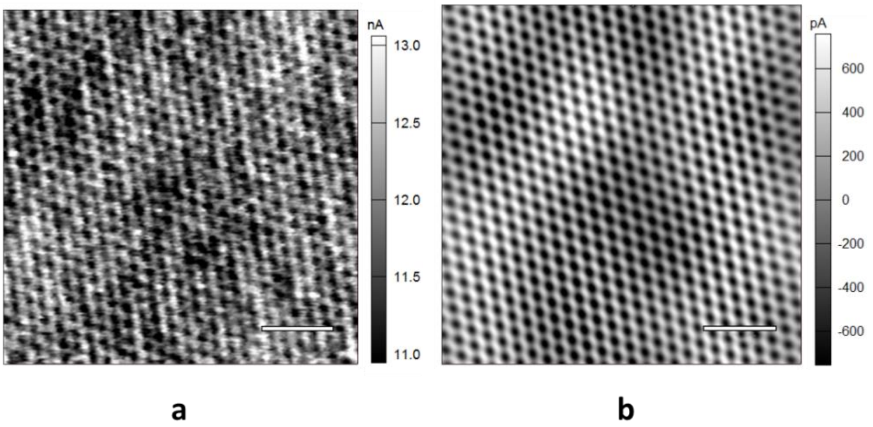

Supplementary Figure 2 | Current mapping of a small area within MoS_2/Gr heterostructure. (a) Before FFT and **(b)** after FFT, scale bar, 5 nm.

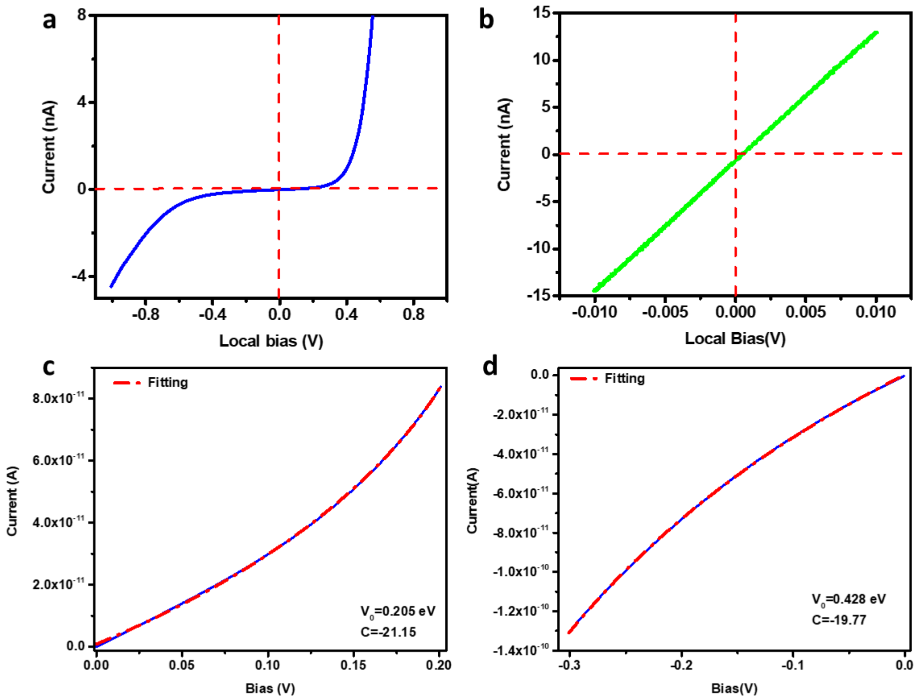

Supplementary Figure 3 | I/V curves and barrier height calculation. (a) I/V curve of 0° twist angle heterojunction. **(b)** I/V curve of graphene. **(c)** and **(d)** Fitting of I/V curves of small positive/negative bias region of Fig.S3a by tunneling model.

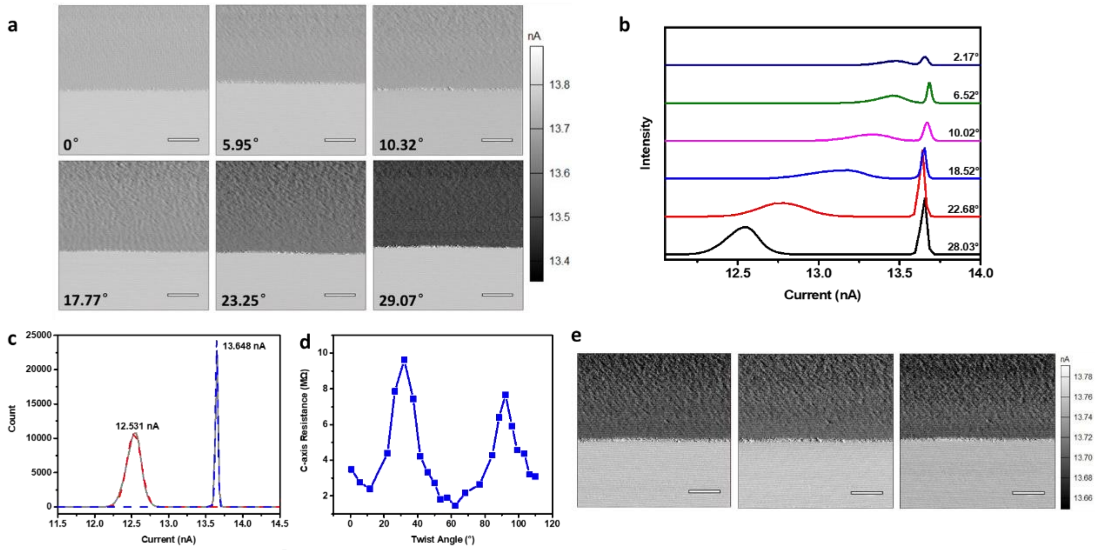

Supplementary Figure 4 | Raw data and repeatability of our experiment. (a) Raw current maps of different twist angles. Scale bars, 100 nm. **(b)** Fig.3**c** before normalization. **(c)** Gauss fitting of Fig. 3**b**. **(d)** Statistic resistances of MoS_2/Gr heterojunctions with different twist angles of another sample. **(e)** 3 consecutive captured current maps of 10.32°, which shows the variation is low, scale bar, 100 nm.

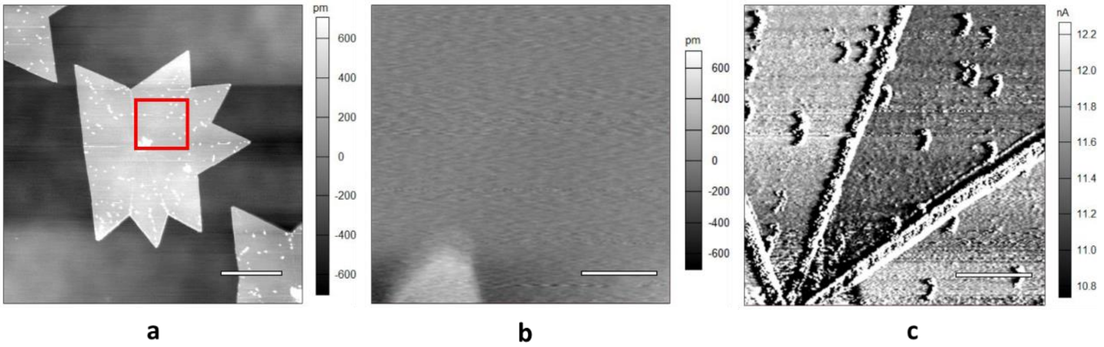

Supplementary Figure 5 | Measurement of a polycrystalline MoS_2 domain on graphene (a) AFM height profile of a polycrystalline MoS_2 sheet on graphene, scale bar, 400 nm. **(b)** and **(c)** are corresponding height and current profile of the red rectangle area in **(a)**, simultaneously captured by the same scanning run, scale bar, 100 nm.

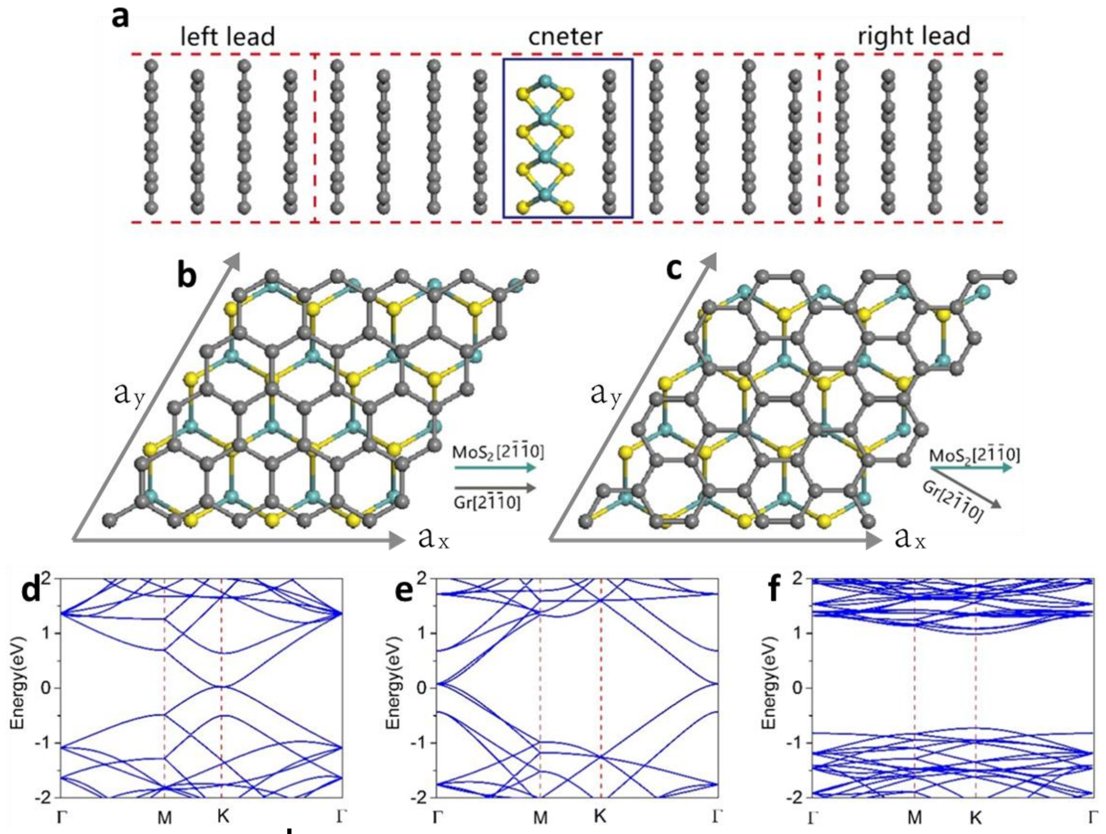

Supplementary Figure 6 | Atomic structures and Band structures of Gr/MoS_2 heterostructures. **(a)** Atomic structure of the transport system when the angle is 0°. **(b)** Atomic structure of graphene and MoS_2 interface when the angle is 0°, which is the part in rectangle box of figure **(a)**. **(c)** Atomic structure of graphene and MoS_2 interface when the angle is 30°. **(d)** Band structure of graphene in 0° system. **(e)** Band structure of graphene in 30° system. **(f)** Band structure of MoS_2.

Supplementary References

1 Myoung, N., Seo, K., Lee, S. J. & Ihm, G. Large current modulation and spin-dependent tunneling of vertical graphene/MoS2 heterostructures. *Acs Nano*, **7**, 7021-7027, (2013).

2 Georgiou, T., R. Jalil, B. D. Belle, L. Britnell, R. V. Gorbachev, S. V. Morozov, Y. J. Kim, A. Gholinia, S. J. Haigh, O. Makarovsky, L. Eaves, L. A. Ponomarenko, A. K. Geim, K. S. Novoselov. & A. Mishchenko. Vertical field-effect transistor based on graphene-WS2 heterostructures for flexible and transparent electronics. *Nat Nanotechnol*, **8**, 100-103, (2013).

3 Ebnonnasir, A., Narayanan, B., Kodambaka, S. & Ciobanu, C. V. Tunable MoS2 bandgap in MoS2-graphene heterostructures. *Applied Physics Letters*, **105**, (2014).
